# The Treatment of Minor Railway Injuries

**Published:** 1895-02

**Authors:** A. L. Hathcock

**Affiliations:** Palestine


### TEXAS MEDICAL JOURNAL.

ESTABLISHED JULY, 1885.

PUBLISHED MONTHLY.—SUBSCRIPTION $2.00 A YEAR.

VOL. X. AUSTIN, FEBRUARY, 1895. NO. 8.

### Original Contributions.

For Texas Medical Journal.

### THE TREATMENT OF MINOR RAILWAY INJURIES.

BY A. L. HATHCOCK, M. D., PALESTINE.

I AM led to write this article by the results that I have occasionally seen of the careless treatment of the smaller injuries that railway employes are liable to. A man gets his fingers "pinched" dropping a coupling pin on them, or gets one or more fingers considerably lacerated and contused by having them caught in making a coupling. He applies to a doctor, who washes off the hand with a solution of bi-chloride of mercury, or some other antiseptic solution, perhaps only allowing the solution to flow over the injured fingers; then dusts the wound with iodoform, aristol, or similar powder, and wraps the finger in antiseptic gauze, cotton and a bandage, and, I suppose, trusts to Providence for the balance. The wound will heal readily with such a dressing, but the probabilities are that within a few days the part will become swollen and painful; the temperature goes up, and upon removal of the dressing we find the wound hot, dry, red and swollen, or pus has already formed and burrowed into the adjoining tissues, often resulting in a very serious condition regarding the future usefulness of the member. Especially is this true of the hand, which can be rapidly damaged by the burrowing of pus along the sheaths of the tendons and along the lymphatics to the palm. This is too frequently the result with such injuries, and it should not be so. It can be avoided, not only in most cases, but I believe in every case that is seen early. Very true, these wounds offer the greatest obstacles to perfect antisepsis. The whole member is perhaps covered with grease, dirt and cinders, ground into the tissues, and in some cases it is impossible to render the wound completely sterile, but I believe that in every case sterilization can be so nearly perfected that the wound is safe from damaging infection. The few microbes that remain are soon disposed of by the living cells of the tissues and the wound heals nicely. This result can not be secured, however, by the method of procedure indicated above. It is not enough to render the wound alone aseptic, but the surrounding skin must also be brought to a condition of approximate asepsis. I say approximate, for I do not believe it is possible by any means at our disposal, to rid entirely the skin with its sebacious and sweat glands of bacteria, but we can destroy the great bulk of them and we then expect the tissue cells to defend the injured part from invasion. The living cells may not be able to cope successfully with a dozen pyogenic cocci, but if we destroy ten of the dozen by our methods of cleanliness, the tissues will be able to get rid of the remaining two.

After thoroughly cleansing the skin and the wound, attention should be directed to the determination of the extent of the injury. The wound should be carefully explored, clots removed and the condition of the bone ascertained. All this requires care, especially with reference to the bone, for a fracture, even of a finger, may be overlooked in badly swollen fingers and a hasty examination.

During the exploration of the wound an antiseptic solution should be allowed to play over the parts, thus utilizing the manipulation necessary for the examination in also cleansing the wound. The solution should reach every recess, and, if it be hot, it will be very efficient in arresting the capillary oozing, which is often quite free in this form of injury. If this is not sufficient to control the hemorrhage, elevation, and pressure made with ice cold compression usually succeed.

If stitches are necessary they are put in now and a piece of protective is laid over the wound, followed by several thicknesses of *antiseptic* gauze, which is itself covered with baked cotton and a sterile bandage applied over the whole.

The special technique employed in the treatment of this class of injuries, and all others as for that matter, is not so important as a thorough understanding and a careful application of the principles of antisepsis and asepsis. The secret of success is cleanliness and attention to details. Unnecessary pain is always to be avoided, but the complaints of the patient must not be allowed to interfere with the proper treatment of the wound. A little ether may be given if the pain is great.

The following methods are practiced at the International and Great Northern railroad hospital at this place, and give very satisfactory results. Bruises and sprains are satisfactorily treated with hot solutions of lead, water and laudanum, applied with cloths kept constantly wet, and, after pain and swelling have subsided, friction with a stimulating liniment. Now and then a badly sprained wrist, ankle, or knee will require a splint or plaster bandage, which should be removed as soon as the pain has ceased, for I believe recovery is sometimes retarded by allowing a sprained joint immovably fixed upon a splint until it has grown stiff. Hæmotomas following contusions should be left alone for two or three weeks, and if by this time the tumor is not perceptibly diminished, the parts are cleansed by the process to be described later on, and a free incision made, the clot scooped out, the sac thoroughly irrigated and antiseptic dressing applied.

### LACERATED AND CONTUSED WOUNDS.

Every lacerated wound that is met with in railway surgery is also a contused wound, sometimes the laceration, sometimes the contusion constituting the greater part of the injury. These are the wounds that show the bad results of improper treatment so often and so clearly that I am surprised to find so many good surgeons disposing of them as mere trifles, and, in their haste, will apply a dressing which has not a claim to antisepsis, or asepsis. I am sure this is, in most cases at least, due to carelessness and not to ignorance. The cases are treated as follows: The surgeon scrubs his hands and forearms with a good stiff brush, using plenty of soap and water, washes off the soap with clean water, cleans the nails, uses the brush again, then washes with alcohol and finally with a solution of bichl. of mercury, 1 to 1000. Note that the brush is used on the fingers after cleansing the accummulations from beneath the nails. This is important. Otherwise the septic matter loosed in the process of removing the subungual dirt is more likely to fall out into the wound than if it had been left undisturbed. Having cleansed the hands thoroughly, a softer brush is used for scrubbing the injured parts. Soap and water are used and the surrounding skin must be scrubbed faithfully, the brush being drawn more gently but firmly over the wound several times in finishing this step of the process. The hairs are shaved off and the soap washed off with clear, boiled, water. Now the surgeon's hands are cleaned again, to rid them of the dirt from the patient's skin in cleansing the wound. After dipping them again in bichloride solution, an assistant irrigates the wounded parts with the same solution, and the surgeon proceeds to ascertain the extent of the injury. The finger explores every part of the wound, enlarging the opening if necessary to admit the finger and permit a thorough search for foreign bodies, pieces of bone, blood clots and the like. It will sometimes be necessary to make a counter-opening to remove a splinter, or other foreign body, or to permit of a further examination. I recall a case in which I dissected up a flap from the dorsal surface of a finger, extending from near the root of the nail to near the second phalangeal joint and removed eight wooden splinters. A piece of pine wood a quarter of an inch in diameter had entered over the ungual phalanx, and, striking the bone, split, and pieces were forced in every direction. The entrance wound was small, and I never suspected the amount of injury until I had made the incision for the purpose of removing a splinter that I could not get at through the entrance wound. I finally stitched the flap in place and the wound healed, but a few weeks later the patient returned with a small opening on one side of the finger, from which there was a slight discharge and in which I found another small splinter. According to the patient's statement this was the seventh splinter he had removed since the finger had healed, making fifteen splinters in all that had been removed. Having determined the exact extent of the injury, and at the same time cleansed the wound, the surgeon next clips off with scissors all fragments of tissue that are clearly devitalized and in some instances it will be better to trim the edges of the wound, else the badly contused margins will slough and prevent primary union. On the other hand it is important to preserve every piece of tissue that there is hope will live.

Spurting vessels are ligated with sterile catgut, or twisted, all oozing controlled by hot antiseptic douching, or by elevation and pressure made with ice cold compresses.

If there is a great deal of contusion, but few, if any sutures are employed, and in any case they are drawn no tighter than is necessary to nicely approximate the edges. As a rule it is unwise to attempt to cover up a gap made by the loss of a considerable amount of tissue by pulling unduly upon the surrounding tissues with sutures. On account of the contusion the parts are much more liable to slough than is the case in other wounds, and failure to unite is the rule where thus treated. In cases where the end of the finger has been almost severed through, the nail being held on by palmar tissue, a single suture passed through the skin at the tip of the finger and carried over the nail and through the skin at the root of the nail and tied, will hold the cut surfaces in perfect contact.

If drainage is necessary, a few strands of sterile catgut or silk will serve the purpose very well. Gauze strips laid in the wound drain the larger wounds perfectly.

The dressing made use of consists of a piece of rubber tissue with holes cut in it to permit discharges to pass through, laid next the wound; over this an abundance of 1-2000 bichloride gauze, a layer of baked cotton, and an aseptic bandage covers the whole.

I am not enthusiastic in the use of "dusting powders" in the treatment of recent wounds. I have never seen any good derived from their use, and they form, with the secretions, a mass, that looks filthy, if it is not so. In infected wounds and in granulating wounds that are somewhat sluggish, I have thought that iodoform is useful, and I do not doubt the efficiency of other powders in selected cases.

This dressing will not be removed for a week, if everything goes well. No hemorrhage, little or no pain, no rise of temperature and no odor tells me to leave the dressing alone. In removing and renewing the dressing, the same precautions are taken as in the first dressing. The outer dressings which have been exposed are removed down to the bichloride gauze, and at this point the surgeon cleanses his hands thoroughly as described above, and then proceeds to remove the balance of the dressing. If it is hard, or sticks, warm sterile water is allowed to flow over it until it is soft and comes off readily. Avoid roughly tearing the dressing off, thus breaking loose the feebly united edges and giving the patient considerable unnecessary pain, which is inexcusable. The parts are cleansed of secretions, stitches removed if the wound has united, or if tension is made upon the stitches by swelling to any great degree, drains removed if there is no longer any indication for them, and the wound with the surrounding parts freely irrigated with hot bichloride solution, 1 to 2000.

In applying the second dressing, the protective may be left off, unless there are granulating surfaces to be covered. Otherwise this dressing is the same as the primary one. It may be left on a week, ordinarily, and in many cases it will be the last dressing necessary.

All instruments are boiled and placed in a solution of carbolic acid, 1 to 40, before being used. Silk is boiled and catgut should be used cautiously, unless it be sterilzed by the surgeon himself, as the so-called sterilized catgut of commerce is generally nothing more nor less than a hot bed for germs. I very rarely use it on this account. My experience with "sterilized" rubber drainage tubes has been the same or worse.

### INFECTED WOUNDS.

Small infected wounds should be curetted well and be dressed antiseptically. Small lacerations or abrasions that have become infected, causing swelling, pain, enlargment of lymphatic glands, and suppuration a little later, usually heal without further difficulty if thoroughly curretted and douched with 1 to 1000 bichloride solution and dressed in the same way as described above. Deeper infected wounds should be curetted, freely irrigated and packed lightly with gauze. I think this is decidedly the best method of dealing with these wounds, but because of the pain entailed in curetting, it is not always practicable. If the patient will not take ether, nor submit to the pain of curetment, then nothing more can be done in this direction. In these cases I have gotten fairly good results from thorough irrigation with bichloride solution, injection of hydrogen peroxide and light packing with iodoform gauze. These are the cases in which iodoform does its best. Perfect cleanliness and good drainage should be secured, an antiseptic dressing applied, and changed every day or every other day, according to the amount of discharge, until suppuration has ceased, when the dressing may be left on several days or a week.

